# “Randomized phase II study of azacitidine ± lenalidomide in higher-risk myelodysplastic syndromes and acute myeloid leukemia with a karyotype including Del(5q)”

**DOI:** 10.1038/s41375-022-01537-w

**Published:** 2022-03-11

**Authors:** Bengt Rasmussen, Gudrun Göhring, Elsa Bernard, Lars Nilsson, Magnus Tobiasson, Martin Jädersten, Hege Garelius, Ingunn Dybedal, Kirsten Grønbaek, Elisabeth Ejerblad, Fryderyk Lorenz, Max Flogegård, Claus Werenberg Marcher, Annette Öster Fernström, Lucia Cavelier, Elli Papaemmanuil, Freja Ebeling, Astrid Olsnes Kittang, Jan Maxwell Nørgaard, Leonie Saft, Lars Möllgård, Eva Hellström-Lindberg

**Affiliations:** 1 School of Medical Sciences, Örebro University, Örebro, Sweden; 2 Department of Human Genetics, Hannover Medical School, Hannover, Germany; 3 Computational Oncology Service, Department of Epidemiology & Biostatistics, Memorial Sloan Kettering Cancer Center, New York, NY USA; 4 Department of Hematology, Oncology and Radiation Physics, Skåne University Hospital, Lund, Sweden; 5 Center for Hematology and Regenerative Medicine, Department of Medicine Huddinge, Karolinska Institutet, Karolinska University Hospital, Stockholm, Sweden; 6 Section for Hematology and Coagulation, Department of Medicine, Sahlgrenska University Hospital, Gothenburg, Sweden; 7 Department of Hematology, Oslo University Hospital, Oslo, Norway; 8 Department of Hematology, Rigshospitalet, Copenhagen University Hospital, Copenhagen, Denmark; 9 Department of Medical Science, Section of Hematology, Uppsala University, Uppsala, Sweden; 10 Department of Medical Biosciences, Umeå University, Umeå, Sweden; 11 Department of Internal Medicine, Falun General Hospital, Falun, Sweden; 12 Department of Hematology, Odense University Hospital, Odense, Denmark; 13 Department of Medicine Huddinge, Center for Hematology and Regenerative Medicine (HERM), Karolinska Institutet, Stockholm, Sweden; 14 Clinical Genomics Facility, Uppsala, Science for Life Laboratory IGP, Rudbeck Laboratory, Uppsala University and Clinical Genetics, Uppsala University Hospital, Uppsala, Sweden; 15 Department of Medicine, Division of Hematology, Helsinki University Central Hospital, Helsinki, Finland; 16 Division for Hematology, Department of Medicine, Haukeland University Hospital, Bergen, Norway; 17 Department of Hematology, Aarhus University Hospital, Aarhus, Denmark; 18 Department of Clinical Pathology, Division of Hematopathology, Karolinska University Hospital and Institute, Solna, Stockholm, Sweden

**Keywords:** Myelodysplastic syndrome, Leukaemia, Chemotherapy

## To the Editor:

The hypomethylating agent azacitidine (AZA) is first-line therapy in higher-risk myelodysplastic syndromes (MDS), and in Europe the only licensed therapy for this MDS category. Lenalidomide (LEN) is an effective treatment for lower-risk MDS with deletion of chromosome 5q (del(5q)) with cytogenetic remission induced in about 45% of patients [1]. LEN selectively induces apoptosis of del(5q) MDS cells through ubiquitination and degradation of *CSNK1A1*, located on chromosome 5 [2, 3], a process dependent on functioning *TP53*. Bernard, et al recently described 378 *TP53*-mutated MDS patients showing that a multiple-hit *TP53* lesion independently predicts poor outcome, while mono-allelic mutational status is more similar to wild-type *TP53* [4]. The direct molecular effect of LEN makes it an interesting AZA candidate partner for higher-risk MDS patients carrying del(5q). Between 2007 and 2009 we performed a phase II study treating 28 patients with higher-risk MDS and secondary acute myeloid leukemia (AML) with chromosome 5 abnormalities with LEN as monotherapy, showing therapeutic response in 35% of patients [5].

In this Nordic MDS group prospective, multicenter, open-label, randomized phase II study, we tested the hypothesis that AZA + LEN is superior to AZA alone in patients with higher-risk MDS (International Prognostic Scoring System Intermediate risk 2 (IPSS INT-2) and High) and low blast AML with multilineage dysplasia and 20–29% blasts and with a karyotype including del(5q). Patients were centrally randomized in blocks in a 1:1 manner to standard dose of AZA 5-2-2 (75 mg/m^2^/day subcutaneously [6]) with a total cycle length of 4 weeks, or AZA + LEN. The initial dose of LEN was 10 mg, oral, daily, 21/28 days, starting day one in each AZA cycle and leaving the last week free of treatment. If tolerated, the dose was escalated to 25 mg daily during cycle four to six.

The primary endpoint was response according to 2006 International Working Group criteria for MDS [7]. Secondary endpoints encompassed cytogenetic response after three cycles and six cycles (fluorescence in situ hybridization (FISH) using the LSI EGR1/D5S23, D5S721 FISH probe), karyotype after six cycles, safety, AZA cycle intervals between groups, mutational status, relapse and survival from the time of randomization to death. All patients underwent annual follow-up from start of treatment until 3 years post-treatment. Morphological bone marrow evaluation and cytogenetic analysis were performed centrally and blinded. Gene mutations, at screening phase, as well as *TP53* mutational status were analyzed after the end of study by deep targeted sequencing [4].

Ninety-one patients from 13 centers in Sweden, Denmark, Norway and Finland, were eligible to be enrolled in the screening phase between March 2012 and January 2017 (Supplementary Fig. 1). The median age of the 72 eligible patients was 71.5 years (range, 35–84 years), 54 (75%) were diagnosed with MDS and 18 (25%) with AML and 11 (15%) received one course of AZA before inclusion. The majority of the cohort (83%) had a complex karyotype and 53 patients (76%) carried a *TP53* mutation, whereof 49 (92%) were multi-hit [4]. Thirty-six patients were randomized to each arm (Supplementary Table 1). Thirty-two patients (44%) terminated therapy prior to protocol plan; 15 patients (42%) in the AZA arm and 17 patients (47%) in the AZA + LEN arm (*P* = 0.64). Reasons encompassed disease progression in 10 patients (6 AZA, 4 AZA + LEN (*P* = 0.50)), adverse events in 18 patients (7 AZA, 11 AZA + LEN (*P* = 0.28)) and subject request in two patients in each arm (Supplementary Table 2).

Seventeen patients with early termination died (6 AZA, 11 AZA + LEN (*P* = 0.165)). Cause of death was disease progression in eight patients, four in each arm, infection in five patients, one in the AZA arm, four in the AZA + LEN arm (*P* = 0.36), CNS hemorrhage in two cases, one in each arm and heart failure in two patients (*P* = 0.49). The overall rate of infections did not differ between the arms, with one exception, eight patients (22%) in AZA + LEN and one patient in AZA had an adverse event grade 1 or 2 unspecified infection (*P* = 0.028) (Supplementary Table 3).

Serious adverse events (SAE) were similar in the two groups (Supplementary Table 4).

Six patients (8%) were withdrawn from study during the pre-treatment period, three in each arm (Supplementary Fig. 1).

Treatment response was analyzed in the intention to treat cohort. Forty-seven of these 72 treated patients (65%) completed three cycles and 40 patients (56%) completed all six cycles. The median length on treatment was 24 weeks in both arms (*P* = 0.87).The 4-week cycle interval could be extended in accordance with the protocol which resulted in additional time with a median of 1.5 weeks (range, 0–7) in the AZA group and 2.5 weeks (range, 0–10) in the AZA + LEN group (*P* = 0.25) (Supplementary Table 2). In the AZA + LEN arm, 7 out of 33 patients (21%) increased the lenalidomide dose to 25 mg/day during cycle four to six.

The overall response rate (ORR) in the treated cohort was 39% for AZA and 44% for AZA + LEN arm (*P* = 0.63) and the corresponding marrow complete remission rate was 17 and 28%, respectively (*P* = 0.086) (Table 1). Four patients (11%) in both arms had a hematological improvement. There was no significant differences in erythroid, neutrophil or platelet responses. Complete cytogenetic response (karyotype) was achieved in four patients (11%) in the AZA arm and seven patients (19%) in the AZA + LEN arm (*P* = 0.18) and a partial cytogenetic response was achieved in two patients (6%) in the AZA arm (*P* = 0.49). Eleven patients received, per protocol, study treatment as a bridge to allogeneic stem cell transplantation (allo-SCT), six in the AZA arm and five in the AZA + LEN arm (*P* = 0.74), with a median of 6.5 months from study enrollment to transplantation. Responding patients had a shorter pre-treatment disease duration than non-responders, 1.6 vs 2.4 months (*P* = 0.048) (Supplementary Table 5). No other pre-treatment variables were significantly associated with ORR.

**Table 1:** Response to treatment.

Variable, No. (%)	Total	AZA	AZA + LEN	AZA *vs*AZA + LEN
	*n* = 72	*n* = 36	*n* = 36	*P*
ORR	30 (42)	14 (39)	16 (44)	0.63
CR	6 (8)	4 (11)	2 (6)	0.67
Marrow CR	16 (22)	6 (17)	10 (28)	0.086
PR	0	0	0	1.0
HI	8 (11)	4 (11)	4 (11)	1.0
No response	42 (58)	22 (61)	20 (56)	0.63
Stable disease	8 (11)	5 (14)	3 (8)	0.71
Failure or treatment interrupted due to AE or subject request	34 (47)	17 (47)	17 (47)	1.0
Cytogenetic CR, final assessment	11 (15)	4 (11)	7 (19)	0.18
Cytogenetic PR, final assessment	2 (3)	2 (6)	0	0.49
Cytogenetic response, CR or PR, 3 cycles (FISH)	30 (42)	13 (36)	17 (47)	0.063
No cytogenetic response	28 (39)	16 (44)	12 (33)	0.51
Allogeneic transplantation	11 (15)	6 (17)	5 (14)	0.74

*AE*  adverse events, *AZA* azacitidine, *CR* complete remission, *FISH* fluorescence in situ hybridization *HI*, hematologic improvement, *LEN* lenalidomide, *ORR* overall response rate, *PR* partial remission.

**Fig. 1:**
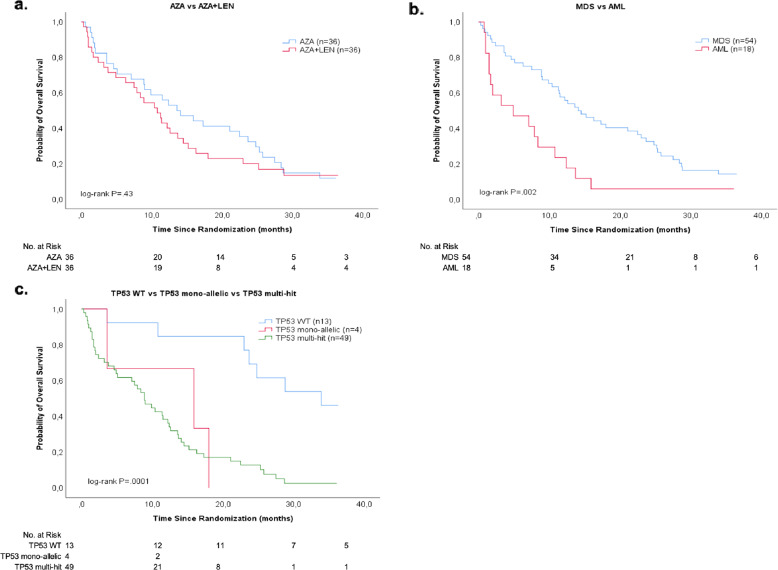
Overall Survival was analyzed by Kaplan-Meier, *P*-values derived from log-rank tests. Number of patients indicated in parentheses. **a** Survival in patients treated with azacitidine (AZA) vs azacitidine + lenalidomide (AZA + LEN) (log-rank *P* = 0.43). **b** Survival comparison between patients with acute myeloid leukemia (AML) or myelodysplastic syndrome (MDS) at inclusion (log-rank *P* = 0.002). **c** Survival among *TP53* mutation subgroups; *TP53* mono-allelic vs multi-hit vs TP53 wild-type (WT) (log-rank *P* = 0.0001).

Thirty-seven different mutations were detected in 70 analyzed patients (Supplementary Table 6). Fifty-three patients (76%) carried *TP53* mutations and 49 (92%) of these were multi-hit [4]. Among patients with multi-hit *TP53* mutations, 15 (31%) patients had one mutation + del(17p), 16 (33%) had one mutation + copy-neutral loss of heterozygosity, and 18 (37%) patients had bi-or tri-allelic mutations (Supplementary Table 1). ORR among patients carrying a *TP53* mutation was 47% (*P* = 0.99). Responding patients had a median variant allele frequency (VAF), at final assessment, of 0% in the single patient in mono-allelic group and 5.7% (0–93%) in the multi-hit group (*P* = 0.21) (Supplementary Table 7). *TP53* (VAF%) following treatment showed an interesting pattern. In 12 of 15 patients with any response, VAF% was significantly reduced (*P* = 0.0001) at 6 months (Supplementary Fig. 2a), with no significant difference of the change in VAF% between arms (*P* = 0.49). A reduction of del(5q) by FISH was observed after cycle three and at final assessment, without significant difference between treatment arms (Supplementary Fig. 2b). Importantly, FISH positivity increased between 3 months and end of study in several patients, indicating that the tumor-inhibiting effect of treatment may be short-lasting. The median follow-up for all patients was 11.5 months (Fig. 1). At follow-up 36 months (range, 0–36 months) after the last patient completed the trial, 60 patients (83%) were dead and 12 (17%) were alive. The median survival was 11.5 months for the entire study population, 13.6 months in the AZA arm and 10.8 months in the AZA + LEN arm (*P* = 0.43). As expected, a diagnosis of AML (*P* = 0.02), *TP53* mutations, any type (*P* = 0.0001), and no response (*P* = 0.047) were associated with shorter overall survival.

Patients with high-risk MDS have an overall dismal prognosis and within this group those with complex karyotype including del(5q) abnormalities and *TP53* mutations have an even worse prognosis [4]. This is to our knowledge the first prospective randomized clinical trial performed in higher-risk MDS with a defined cytogenetic lesion. The study cohort recruited from Nordic university hospitals showed a high degree of risk factors; high age, AML at diagnosis, marrow fibrosis, complex karyotype, therapy-related disease and two thirds carrying multi-hit *TP53* mutations. Without doubt, this negatively affected response rates and survival. It also affected the rate of patients failing inclusion criteria due to rising bone marrow blast counts and the rate of patients withdrawn from study due to severe adverse events or frank progression. Nevertheless, an important advantage of this study is that our cohort probably is representative for the particular patient population.

Even if the addition of LEN to AZA did not improve the outcome of del(5q) myeloid malignancies with extreme high-risk features, important lessons can be learned from this study. First, it is important to design prospective studies in order to enable detailed assessment of the biological characteristics in defined patient groups undergoing targeted treatment. Secondly, corroborating the findings of other investigators, hypomethylating agent has indeed an anti-tumor effect on *TP53* mutated cells, but it is short-lasting and do not translate into improved patient outcomes. Studies aiming to assess the additional effect of even shorter LEN exposure times may be a relevant next step. We conclude that future therapeutic studies of complex karyotype, *TP53* mutated MDS should be of shorter duration, with molecular follow-up after each cycle, and with a more rapid planning to proceed to allo-SCT, if possible.

## Supplementary information

Supplementary Material

Supplementary tables and figures
