# Supplementary material for: “Randomized phase II study of azacitidine ± lenalidomide in higher-risk myelodysplastic syndromes and acute myeloid leukemia with a karyotype including Del(5q)”
Source: Leukemia. 2022 Mar 11;36(5):1436–9. doi: 10.1038/s41375-022-01537-w (PMC9061286; doi:10.1038/s41375-022-01537-w)
Supplement: Supplementary file 1 — Supplementary Material [file 41375_2022_1537_MOESM1_ESM.pdf]

**Supplementary Material**

# Randomized Phase II Study of Azacitidine ± Lenalidomide in Higher-Risk Myelodysplastic Syndromes and Acute Myeloid Leukemia with a Karyotype Including Del(5q)

## Methods

*Patients* who met the inclusion criteria: (i) MDS with IPSS Int-2 or High or AML with multilineage dysplasia and 20-29% blasts (former refractory anemia with excess blasts in transformation) with a karyotype including del(5q), assessed centrally; (ii) ≥ 18 years of age; (iii) women of childbearing potential must have had a negative pregnancy test prior starting LEN, using adequate contraceptive methods during LEN treatment; (iv) males had to use barrier contraception with women of childbearing potential while on LEN treatment and 28 days after the last dose of LEN; and (v) signed informed consent.

Refractory and relapsed patients could be included as long as they fulfilled the inclusion criteria.

During the first 23 months of the study five of 32 patients who were subjects to screening increased their blasts counts to ≥ 30% between the local and the subsequent screening bone marrow sample, and were thus excluded from the study. This was a problem since primary hospitals were invited to identify these rare patients and refer them to a study center. We experienced that the cohort subject to the study had a more progressive nature than higher-risk MDS patients in general. An amendment was therefore approved by the IRB allowing one cycle of AZA between the diagnostic and the screening bone marrow sample.

Prior treatment with one cycle of AZA was allowed after contact with study centre in cases where the physician determined that the patient was in need of treatment before randomization process was completed. This azacitidine treatment was not counted as one of the six cycles in the study protocol.

*Patients* exclusion criteria were: (a) eligible for upfront allo-SCT without prior induction chemotherapy or AZA; (b) pregnant or lactating females; (c) prior therapy with > 1 cycle of AZA; (d) prior therapy with LEN; (e) expected survival less than 2 months; (f) acute promyelocytic leukemia; (g) central nervous system leukemia; (h) serum creatinine >2.0 mg/dL, serum aminotransferase or alanine transferase >3.0 x upper limit of normal or serum total bilirubin >1.5 mg/dL; (i) prior allergic reaction to thalidomide; or (j) uncontrolled systemic infection.

The sample size was 36 patients for each treatment group, this to compensate for the study population with a high degree of risk factors which could affect the number of patients reaching final assessment.

Nineteen patients (20.9%) were excluded; patients did not fulfil the inclusion criteria due to high blast count (≥30%) (8), no detectable del(5q) (6), centrally revised IPSS score INT-1 (2), clinical judgement (2), and one patient withdrew consent (1).

Patients were centrally randomized in blocks (randomization list was generated using www.randomization.com) in a 1:1 manner to standard dose of AZA 5-2-2 (75 mg/m$^2$/day subcutaneously[6] with a total cycle length of 4 weeks, or AZA+LEN.

The initial dose of LEN was 10 mg, daily, 21/28 days. This mode of dosing was evaluated in Möllgård *et al*[(5)] phase II study with LEN as mono-therapy in high-risk MDS and secondary AML and found feasible. In order to increase the cytoreductive effect, we considered to escalate the dose to 20 mg, daily, 21/28 days, during cycle four to six. The dose escalation was changed to 25 mg, daily, after discussion with Celgene.

The study was approved by National Ethical committees in Sweden, Denmark, Norway and Finland, carried out in accordance with the Declaration of Helsinki/Tokyo/Venice[(8)] and registered at www.clinicaltrials.gov: NTC01556477. Patients provided written informed consent. The study was financed by the Nordic MDS group, Celgene contributed with a research grant and study drug (lenalidomide).

**Dose modification and discontinuation of drugs**

*Hematological toxicity*

*Azacitidine arm*

Dose modifications due to hematologic toxicity (thrombocytopenia and/or neutropenia) were performed based on the white blood cell (WBC) or platelet (plt) counts at start of treatment.

If WBC >3.0 x$10^9$/L and absolute neutrophil count (ANC) >1.5 x$10^9$/L, and plt >75 x$10^9$/L at start of treatment, a hematological toxicity with an ANC count of <0.5 x$10^9$/L or a plt count of <20 x$10^9$/L led to an increase in dose interval. Intervals between AZA cycles increased to maximum of 8 weeks. If persistent significant cytopenia after 6

weeks, a bone marrow was taken to differentiate between bone marrow hypoplasia due to AZA and disease progression.

If WBC <3.0 x10$^{9}$/L or ANC <1.5 x10$^{9}$/L or plt <75 x10$^{9}$/L at start of treatment, the treatment started without delay if the decreased in WBC or ANC or platelets from that prior to treatment was ≤ 50% or >50% but with an improvement in any cell line differentiation or if ANC was ≥0.5 x10$^{9}$/L and plt ≥20 x10$^{9}$/L.

*Azacitidine plus lenalidomide arm*

Dose modifications due to hematologic toxicity (thrombocytopenia and/or neutropenia) were performed based on the WBC or plt counts at start of treatment. If clinical significant cytopenia, compared to the baseline levels, occurred, LEN was interrupted (during days 1-21). If WBC >3.0 x10$^{9}$/L and ANC >1.5 x10$^{9}$/L, and plt >75 x10$^{9}$/L at start of treatment, a hematological toxicity with an ANC count of <0.5 x10$^{9}$/L or a plt count of <20 x10$^{9}$/L led to dose interruption, until recovery above those thresholds. If recovery toke place before day 21, LEN was resumed at the same dose level if the patient was on 10 mg or lowered the dose to 10 mg if the patient was on 25 mg. At resumption of LEN treatment during day 1-21, the patient proceed to day 21 without addition of extra days of LEN treatment. If acceptable levels of platelets and/ or neutrophils were not reached, the next AZA cycle was delayed as in AZA arm to a maximum of 8 weeks. Intervals between AZA cycles increased to maximum of 8 weeks. If persistent significant cytopenia after 6 weeks, a bone marrow was taken to differentiate between bone marrow hypoplasia and disease progression. If WBC <3.0 x10$^{9}$/L or ANC <1.5 x10$^{9}$/L or plt <75 x10$^{9}$/L at start of treatment, the treatment started without delay if the decreased in WBC or ANC or platelets from that prior to

treatment was ≤ 50% or > 50% but with an improvement in any cell line differentiation or if ANC was ≥0.5 x$10^9$/L and plt ≥20 x$10^9$/L. If not, this led to dose delay, as above. The dose of LEN was not increased to 25 mg if the interval between AZA cycles had been increased for more than 5 weeks.

*Concomitant therapy*

Other active treatment for MDS, including steroids, other than low permanent doses (≤ 25 mg oral prednisolone) for inflammatory disorders, was not allowed during the study. G-CSF was allowed, with doses according to investigators judgement, in case of neutropenia with infection, or if ANC <0.5 x $10^9$/L. Prophylactic medication was used according to local routines. Erythropoietin was not allowed during the study.

**Withdrawal from study**

The following events were considered sufficient reasons for discontinuing a patient from the study medication: (a) adverse event(s) that, in the judgement of the investigator, might cause severe or permanent harm or which ruled out continuation of study medication: (b)  patient withdrawn consent: (c) patient lost to followed-up: (d) death: (e) protocol violation: (f) overt disease progression. Responding patients, who were eligible for allo-SCT, could exit the study after cycle three, four or five and then be subject for end-of-study assessment.

**Long-term follow-up**

All patients underwent follow-up once yearly from start of treatment until after three years. Follow-up included survival, AML transformation, and MDS-specific treatment.

**Outcomes and investigations**

Response was assessed by two independent observers after six cycles of AZA or AZA+LEN treatment, or at the end of protocol treatment if this occurred at an earlier time point.

Bone marrow morphology and histopathology (trephines biopsies) were analyzed centrally and blinded at inclusion and at final assessment (Karolinska University Hospital, Solna, Department of Pathology Hematopathology Lab). The percentage of blasts was calculated in bone marrow smears and/or imprints and correlated to the percentage of blasts in biopsies by using CD34 immunohistochemistry.

**Cytogenetic response**

Cytogenetic analysis by karyotyping was analyzed centrally and blinded (Department of Human Genetics, Hannover Medical School). Cytogenetic response was assessed after six cycles of treatment. Del(5q) was confirmed by FISH on bone marrow fixed cells and bone marrow slides, after three and six cycles. FISH was performed in order to detect the del(5q), especially in cases with a complex karyotype, and as a quantitative method to analyze the clone size. Karyotype without FISH was sufficient to categorize the patient. At inclusion, an abnormal clone could be identified by less than 15 metaphases. During follow-up, less than 15 metaphases were adequate if a known clonal aberration was detected again. Less than 15 metaphases without aberrations during follow-up were not sufficient, unless FISH was available and del(5q) was identified in the major clone.

Complete cytogenetic response: disappearance of the 5q deletion and any other chromosome aberration.

Partial cytogenetic response: reduction of aberrant cells of more than 50% compared to the previous cytogenetic investigation.

No cytogenetic response: persistent clones or reduction of aberrant cells of less than 50%.

**Next generation sequencing**

Mononuclear cells or CD34+ cells from bone marrow and peripheral blood were isolated by using Lymphoprep™ and genomic DNA was separated from these cells by using GeneElute DNA extraction kit (Sigma Aldrich). Following failure of initial sequencing six patients were analyzed by TruSight™ Myeloid Sequencing panel and two of these patients were also analyzed by Twist Bioscience™ Myeloid panel. To evaluate the effect of treatment cryopreserved separated MNC or CD34+ cells were analyzed by TruSight™, to analyze the presence and size of residual mutations. Variant allele frequency (VAF) was analyzed in all samples.

**Statistical analysis**

This phase II study was designed to detect an improvement in efficacy of ≥20%, as defined as ≥20% of patients reaching the primary endpoint. We used the Simon Two-Stage Adaptive Design. With an unacceptable response probability $p_0$=0.10 and an acceptable response probability $p_1$=0.30, with a $\alpha$=0.05 and a power=0.90, the sample size was 35 for each group. In the case, the first stage consisted of 18 patients and if there was up to two responses the trial was terminated; otherwise, accrual continued to a total of 35 patients. This scenario implied two cohorts of 35 patients (70 in total). The randomization process was not stratified by any patient characteristics.

Continuous data were described by mean and median (range) values depending on the distribution of data. $\chi^2$- or Fisher´s exact tests were used to measure the difference between responders and non-responders. Continuous variables regarding mutational status, cycle interval and response were used univariate analysis by a Mann-Whitney *U* test or T-test independent analysis. *TP53* VAF% changes were analysed by paired *t*-test. Survival estimates were calculated by the Kaplan-Meier method, and tests for differences in survival were done using log-rank tests. Survival was calculated from date of randomization to death, censoring date was the date patient was last known to be alive.

## Results

### Patients´ Characteristics

Thirty (42%) patients were female. Median marrow blast percentage was 7% for MDS patients and 21.5% for AML patients and 18 patients (25%) had marrow fibrosis, grade 2 or 3. Sixty-two patients (86%) were transfusion dependent and five (7%) had been treated with induction chemotherapy, whereof three had MDS-AML and two had *de novo* AML. According to IPSS, the cytogenetic risk group was good in eight patients (11%), intermediate in four patients (6%) and poor in 60 patients (83%). Baseline data were similar between the two treatment arms except that patients with t-MDS were more frequent in AZA+LEN arm (31%), vs 11% in the AZA arm (*P*=0.029) (Supplementary Table 1).

### Adverse events

Adverse events were evaluated in the intention to treat cohort (Supplementary Fig. 1).

One hundred and eighty-eight SAE´s in 54 patients (75%) were reported. The most frequent criterion being hospitalization. Seventeen patients had ≥5 SAE´s, which accounted for 60% of the total SAE´s. No suspected unexpected serious adverse reaction was observed.

**Treatment response**

The mean interval following cycle 2 was 4.2 weeks in the AZA arm and 4.7 in the AZA+LEN arm (*P*=0.048) due to more frequent per protocol dose modifications; nine patients (35%) in the AZA+LEN arm and one patient (4%) in the AZA arm (*P*=0.005) were subject to dose modification (Supplementary Table 2).

**Mutational status**

The median number of mutations was two (range 0-5) in the AZA arm and two (range, 0-6) in the AZA+LEN arm (Supplementary Table 1). Ten patients (14%) had *SF3B1* mutations, 12 (17%) *DNMT3A* mutations and six (9%) *ASXL1* mutations (Supplementary Table 6). There was no significant difference in ORR according to mutational status (Supplementary Table 5).

**Follow-up and survival**

Survival was analysed in the intention to treat cohort (72 patients) (Supplementary Fig. 1).

Twenty-seven (38%) patients received subsequent treatment, 11 patients (31%) in AZA arm and 16 patients (44%) in the AZA+LEN arm (*P*=0.21). Responding patients

in both arms were per protocol allowed to continue with AZA after six cycles according to standard practice and 18 patients (25%) continued with AZA, nine patients in each arm. Other subsequent treatments encompassed donor lymphocyte infusion in two (3%) patients relapsing after allo-SCT, hydroxyurea in four (6%) patients, induction chemotherapy in four (6%) patients, tioguanine in three (42%) patients and low dose cytarabine, rigosertib, decitabine, venetoclax, radiotherapy or single lenalidomide in one (1%) patient each.

Twelve patients were treated with allo-SCT, nine patient after a response to treatment, two patients with a stable disease and one patient after the AZA+LEN treatment was stopped due to subject request. This patient continued with AZA and was treated with allo-SCT after five months. The median survival was 21.1 months for responding patients treated with allo-SCT and 14.5 months for responding patients not treated with allo-SCT (*P*=0.92).

## References

8. World Medical Association Declaration of Helsinki. Ethical principles for medical research involving human subjects. Nurs Ethics. 2002;9(1):105-9.
