# Supplementary material for: “Randomized phase II study of azacitidine ± lenalidomide in higher-risk myelodysplastic syndromes and acute myeloid leukemia with a karyotype including Del(5q)”
Source: Leukemia. 2022 Mar 11;36(5):1436–9. doi: 10.1038/s41375-022-01537-w (PMC9061286; doi:10.1038/s41375-022-01537-w)
Supplement: Supplementary file 2 — Supplementary tables and figures [file 41375_2022_1537_MOESM2_ESM.pdf]

Supplementary Table 1. Baseline Characteristics

Factor	All included patients n=72	AZA n=36	AZA+LEN n=36	AZA vs AZA+LEN *P*
Age, years, median	71.5 (35-84)	73 (35-82)	70,5 (37-84)	0.54
Female	30 (42)	16 (44)	14 (39)	0.63
RCMD	12 (17)	6 (17)	6 (17)	1
RCMD-RS	2 (3)	0	2 (6)	0.49
RAEB-1	16 (22)	9 (25)	7 (19)	0.57
RAEB-2	24 (33)	14 (39)	10 (28)	0.32
tMDS	15 (21)	4 (11)	11 (31)	0.029
AML de novo	0	0	0	
AML de novo relapse	1 (1)	1 (3)	0	1.0
AML-MDS	17 (24)	6 (17)	11 (31)	0.17
Marrow blasts, MDS	7.0 (0-19)	7.0 (0-19)	6.5 (1-18)	0.77
Marrow blasts, AML	21.5 (1-30)	22.0 (4-28)	21.5 (1-30)	0.66
Marrow cellularity, MDS	75 (25-100)	70 (25-100)	80 (30-100)	0.83
Marrow cellularity, AML	70 (10-100)	65 (10-80)	80 (30-100)	0.078
Marrow firbosis, grade 2 or 3	18 (25)	5 (14)	13 (36)	0.066
TP53 mutation present	53 (74)	27 (75)	26 (72)	0.89
TP53 mutation mono-allelic	4 (6)	2 (6)	2 (6)	1.0
TP53 mutations multi-hit	49 (68)	25 (69)	24 (67)	0.91
TP53 1 mutation + del	15 (21)	10 (28)	5 (14)	0.18
TP53 1 mutation + cnloh	16 (22)	8 (22)	8 (22)	0.90
TP53 >1 mutations	18 (25)	7 (19)	11 (31)	0.22
TP53, WT	13 (18)	8 (22)	5 (14)	0.42
Number of mutations, median	2 (0-6)	2 (0-5)	2 (0-6)	0.47
Comorbidity Solid tumor, past	17 (24)	10 (28)	7 (19)	0.44
Hematological malignacies Past	10 (14)	3 (8)	7 (19)	0.19
Hematological malignacies Current	2 (3)	0	2 (6)	0.24
Prior chemotherapy	19 (26)	7 (19)	12 (33)	0.16
Prior radiation	6 (8)	1 (3)	5 (11)	0.11
Prior transplant	2 (3)	1 (3)	1 (3)	1.0
Prior treatment				
No previous treatment	43 (60)	20 (56)	23 (64)	0.30
Induction chemotherapy	5 (7)	3 (8)	2 (6)	1.0
ESA	11 (15)	8	3	0.12
One course of Azacitidine	11 (15)	5 (14)	6 (17)	0.74
Cytogenetics, IPSS good	8 (11)	1 (3)	7 (19)	0.06
Cytogenetics, IPSS intermediate	4 (6)	4 (11)	0	0.12
Cytogenetics, IPSS poor	60 (83)	31 (86)	29 (81)	0.53
Cytogenetics IPSS-R Good	11 (15)	4 (11)	7 (19)	0.33
Cytogenetics IPSS-R Intermediate	1 (1)	1 (3)	0	1.0
Cytogenetics IPSS-R Poor	6 (8)	2 (6)	4 (11)	0.67
Cytogenetics IPSS-R Very poor	54 (75)	29 (81)	25 (69)	0.28
IPSS Low + Int-1	6 (8)	3 (8)	3 (8)	1.0
IPSS Int-2	30 (41)	14 (39)	16 (44)	0.25
IPSS High	18 (25)	12 (33)	6 (17)	0.18
IPSS-R Low + Intermediate	6 (8)	3 (8)	3 (8)	1.0
IPSS-R High	14 (19)	6 (17)	8 (22)	0.34
IPSS-R Very high	34 (47)	20 (56)	14 (39)	0.33
HgB (g/dL)	9.2 (6.2-12.0)	9.3 (6.2-12.0)	9.0 (6.9-11.8)	0.66
ANC x 10^9/L	1.00 (0.10-20.60)	0.95 (0.10-20.60)	1.05 (0.20-6.70)	0.47
Platelets x 10^9/L	43 (4-252)	39.5 (5-211)	57 (4-252)	0.43
Transfusion dependent at inclusion	62 (86)	32 (89)	30 (83)	0.69

NOTE: Continuous variables (min-max) and caregorical variables (%) are reported.

Abbreviations: AML, acute myeloid leukemia; ANC, absolute neutrophil count; AZA, azacitidine; cnloh, copy-neutral loss of heterozygosity; del, deletion; ESA, erythropoiesis stimulating agent; HgB, hemoglobin; Int, intermediate; IPSS, International Prognostic Scoring System; IPSS-R, Revised-IPSS; LEN, lenalidomide; MDS, myelodysplastic syndrome; RAEB, refractory anemia with excess blasts; RCMD, refractory cytopenia with multilineage dysplasia; RS, ring sideroblasts; t, treated; WT, wild type.

Supplementary Table 2. azacitidine cycle intervals

Variable, No. (%) or Mean	ITT Total n=72	ITT AZA n=36	ITT AZA+LEN n=36	ITT AZA *vs* AZA+LEN *P*	Treated cohort Total n=66	Treated cohort AZA n=33	Treated cohort AZA+LEN n=33	Treated cohort AZA *vs* AZA+LEN *P*
Stopped AE	18 (25)	7 (19)	11 (31)	0.28	16 (25)	6 (18)	10 (38)	0.25
Stopped disease progression	10 (14)	6 (17)	4 (11)	0.50	8 (12)	6 (18)	2 (6)	0.26
Dose interrupted - toxicity or complictions, total					38 (58)	17 (52)	21 (64)	0.32
Dose interrupted - per protocol dose modification, total					23 (35)	9 (27)	14 (42)	0.20
Dose interrupted - non protocol dose modification, total					25 (38)	10 (30)	15 (45)	0.21
Cycle 1, (n=66/33/33, total/AZA/AZA+LEN)								
Number of weeks , cycle 1 (mean)					4.9	4.8	5.0	0.65
Dose interrupted - toxicity or complictions, cycle 1					22 (33)	9 (27)	13 (39)	0.30
Dose interrupted - per protocol dose modification, cycle 1					12 (18)	6 (18)	6 (18)	1.0
Dose interrupted - non protocol dose modification, cycle 1					5 (8)	1 (3)	4 (12)	0.36
Cycle 2, (n=53/27/26, total/AZA/AZA+LEN)								
Number of weeks , cycle 2 (mean)					4.5	4.2	4.7	**0.048**
Dose interrupted - toxicity or complictions, cycle 2					9 (17)	3 (11)	6 (23)	0.29
Dose interrupted - per protocol dose modification, cycle 2					10 (19)	1 (4)	9 (35)	**0.005**
Dose interrupted - non protocol dose modification, cycle 2					6 (11)	2 (7)	4 (15)	0.42
Cycle 3, (n=49/25/24, total/AZA/AZA+LEN)								
Number of weeks , cycle 3 (mean)					4.3	4.3	4.4	0.72
Dose interrupted - toxicity or complictions, cycle 3					8 (16)	3 (12)	5 (21)	0.46
Dose interrupted - per protocol dose modification, cycle 3					4 (8)	2 (8)	2 (8)	1.0
Dose interrupted - non protocol dose modification, cycle 3					6 (12)	0	6 (25)	**0.010**
Cycle 4, (n=45/23/22, total/AZA/AZA+LEN)								
Number of weeks, number of cycle 4 (mean)					4.3	4.2	4.3	0.70
Dose interrupted - toxicity or complictions, cycle 4					5 (11)	4 (17)	1 (5)	0.35
Dose interrupted - per protocol dose modification, cycle 4					5 (11)	1 (4)	4 (18)	0.19
Dose interrupted - non protocol dose modification, cycle 4					10 (22)	2 (9)	8 (36)	**0.035**
Cycle 5, (n=38/19/19, total/AZA/AZA+LEN)								
Number of weeks , cycle 5 (mean)					4.3	4.3	4.3	0.87
Dose interrupted - toxicity or complictions, cycle 5					6 (16)	2 (11)	4 (21)	0.66
Dose interrupted - per protocol dose modification, cycle 5					3 (8)	0	3 (16)	0.23
Dose interrupted - non protocol dose modification, cycle 5					5 (13)	1 (5)	4 (21)	0.34
Cycle 6, (n=34/18/16, total/AZA/AZA+LEN)								
Number of weeks , cycle 6 (mean)					4.2	4.1	4.3	0.62
Dose interrupted - toxicity or complictions, cycle 6					2 (6)	0	2 (13)	0.21
Dose interrupted - per protocol dose modification, cycle 6					7 (21)	1 (6)	6 (38)	**0.035**
Dose interrupted - non protocol dose modification, cycle 6					8 (24)	6 (33)	2 (13)	0.23

Abbreviations: AE, adverse event; AZA, azacitidine; ITT, intention to treat; LEN, lenalidomide; ORR, overall response rate.

Supplementary Table 3. Most Common Adverse Events in the Study Population

Event	AZA (n=36) Any Grade	AZA+LEN (n=36) Any Grade	AZA *vs* AZA+LEN
	number of patients (percent)		*P*
**Hematologic adverse event**			
Neutropenia	26 (72)	29 (81)	0.41
Anemia	26 (72)	29 (81)	0.41
Thrombocytopenia	21 (58)	28 (78)	0.077
Febrile neutropenia	11 (31)	15 (42)	0.33
**Nonhematologic adverse event**			
Constipation	8 (22)	13 (36)	0.20
Pneumonia	9 (25)	9 (25)	1.0
Nausea	3 (8)	9 (25)	0.058
Infection, unspecified	1 (3)	8 (22)	**0.028**
Fever	6 (17)	7 (19)	0.76
Injection site reaktion	7 (19)	6 (17)	0.76
Diarrhoea	5 (14)	7 (19)	0.53
Rash	4 (11)	7 (19)	0.33
Sepsis	3 (8)	6 (17)	0.48
Urinary tract infection	6 (17)	2 (6)	0.26

Adverse events in more than 15% of patients in either treatment group.
Abbrevations: AZA, azacitidine; LEN, lenalidomide.

Supplementary Table 4. Most Common Serious Adverse Events in the Study Population

Event	AZA (n=36) Grade 3 or 4	AZA+LEN (n=36) Grade 3 or 4	AZA *vs* AZA+LEN
	number of patients (percent)		*P*
**Hematologic adverse event**			
Severe myelosuppression	28 (78)	31 (86)	0.36
Neutropenia	24 (67)	27 (75)	0.44
Anemia	13 (36)	16 (44)	0.47
Thrombocytopenia	18 (50)	25 (69)	0.093
Febrile neutropenia	11 (31)	15 (42)	0.33
**Nonhematologic adverse event**			
Pneumonia	8 (22)	7 (19)	0.78
Infection	1 (3)	5 (14)	0.20
Fever	2 (6)	4 (11)	0.67
Diarrhoea	2 (6)	1 (3)	1.0
Rash	1 (3)	2 (6)	1.0
Sepsis	3 (8)	6 (17)	0.48
Heart failure	0	4 (11)	0.12
Fungal infection	3 (8)	0	0.24
Subarachnoidal bleeding cerebral hematoma	0	3 (8)	0.24
Acute subdural hematoma	2 (6)	1 (3)	1.0
Perianal infection	0	2 (6)	0.49
Pulmonary mucosis aspergillosis	0	2 (6)	0.49
Sweets syndrome	2 (6)	0	0.49

Serious Adverse events in more than 5% of patients in either treatment group.
Abbrevations: AZA, azacitidine; LEN, lenalidomide.

Supplementary Table 5. Pre-treatment variables associated with response (ORR)

Variable, No. (%) or Median (range)		Total n=66	Response n=30	No response n=36	Response *vs* No response *P*	Odds ratio	CI 95%
Age		70.5 (35-84)	69.5 (35-84)	73 (52-83)	0.15		
Gender, female		28 (42)	12 (43)	16 (57)	0.72	1.20	0.45-3.21
Disease duration, months		2.2 (0-170.6)	1.6 (0-170.6)	2.4 (0.3-108.4)	**0.048**		
Marrow blasts %		8 (0-30)	8 (0-30)	8 (1-24)	0.86		
Cellularity %		70 (10-100)	70 (30-100)	70 (10-100)	0.83		
Fibrosis, grade 2 or 3		16 (24)	5 (31)	11 (69)	0.066	3.06	0.90-10.33
Comorbidity							
MDS-CI Low		37 (56)	15 (41)	22 (59)	0.37	1.57	0.59-4.19
MDS-CI Intermediate		23 (35)	13 (57)	10 (44)	0.19	0.50	0.18-1.40
MDS-CI High		6 (9)	2 (33)	4 (67)	0.68	1.75	0.30-10.29
Hematological malignacies Past		9 (14)	4 (44)	5(56)	1.0	1.05	0.26-4.31
No prior treatment		39 (59)	20 (67)	19 (53)	0.25	0.56	0.21-1.52
Prior treatment, Induction		5 (8)	1 (20)	4 (80)	0.36	3.74	0.40-35.47
Prior treatment ESA		11 (17)	6 (55)	5 (45)	0.51	0.65	0.18-2.37
Prior radiation		5 (8)	1 (20)	4 (80)	0.26	3.63	0.38-34.33
MDS		51 (77)	25 (49)	26 (51)	0.28	0.52	0.16-1.74
WHO subgroup RCMD		11 (17)	3 (27)	8 (73)	0.19	2.57	0.62-10.73
WHO subgroup RCMD-RS		2 (3)	1 (50)	1(50)	1.0	0.83	0.050-13.83
WHO subgroup RAEB 1		15 (23)	7 (47)	8 (53)	0.92	0.94	0.30-2.98
WHO subgroup RAEB 2		23 (35)	14 (61)	9 (39)	0.066	0.38	0.14-1.08
tMDS		14 (21)	5 (36)	9 (64)	0.24	2.19	0.59-8.04
AML		15 (23)	5 (33)	10 (66)	0.28	1.92	0.58-6.42
HgB level g/dL		9.1 (6.2-12.0)	9.3 (6.2-11.8)	9.1 (7.2-12.0)	0.58		
ANC, x 10^9/L		1.0 (0.1-20.6)	0.95 (0.1-12.8)	1.1 (0.1-20.6)	0.94		
Platelets, x 10^9/L		46 (5-252)	59 (10-222)	42 (4-252)	0.12		
Transfusion dependent	Yes	57 (86)	24 (42)	33 (58)	0.28	2.75	0.63-12.11
	No	9 (14)	6 (67)	3 (33)			
IPSS cytogenetic risk group	Favorable	8 (12)	5 (63)	3 (37)	0.45	0.46	0.099-2.08
	Intermediate	4 (6)	0	4 (100)	0.12		
	Adverse	54 (82)	25 (46)	29 (54)	0.77	0.83	0.23-2.94
IPSS risk score	Int-1	6 (12)	2 (33)	4 (67)	0.67	2.09	0.35-12.59
	Int-2	27 (53)	12 (44)	15 (58)	0.49	1.48	0.49-4.46
	High	18 (27)	11 (61)	7 (39)	0.20	0.47	0.15-1.51
Cytogenetics	5q- + 0 abn	7 (11)	5 (71)	2 (28)	0.23	0.29	0.053-1.64
	5q- + 1 abn	5 (8)	0	5 (100)	0.058		
	5q- + 2 or more	54 (82)	25 (46)	29 (54)	0.77	0.83	0.23-2.94
BM FISH 5q31 %		76 (9-98)	76 (17-96)	74 (9-98)	0.41		
Number of mutations		2 (0-6)	2 (0-4)	2 (0-6)	0.26		
TP53 mutation		47 (71)	22 (47)	25 (53)	0.99	1.01	0.33-3.07
TP53 mutation, mono-allelic		4 (9)	1 (25)	3 (75)	0.62	2.81	0.28-28.53
TP53 mutation, multi-allelic		43 (91)	21 (49)	22 (51)	0.65	0.79	0.28-2.25
TP53 WT		13 (20)	6 (46)	7 (55)	0.95	1.03	0.31-3.52
TP53 mutation, VAF%		0.497 (0.092-0.938)	0.577 (0.112-0.920)	0.449 (0.092-0.938)	0.69		

Note: Odds ratios of responders compared with non-responders.

Abbreviations: abn, abnormality; AML, acute myeloid leukemia; ANC, absolute neutrophil count; Aza, azacitidine; BM, bone marrow; CI, comorbidity index; ESA, erythropoiesis stimulating agent; FISH, fluorescence in situ hybridization; HgB, hemoglobin; Int, intermediate; IPSS, International Prognostic Scoring System; IPSS-R, Revised-IPSS; Len, lenalidomide; MDS, myelodysplastic syndrome; RAEB, refractory anemia with excess blasts; RCMD, refractory cytopenia with multilineage dysplasia; RS, ring sideroblasts; t, treated; VAF, variant allele frequency, WT, wild type.

Supplementary Table 6. Mutations NGS

Mutation	Incidence, NGS n=70 (%)	No. Mutated AZA, AZA+LEN
ASXL	6 (9)	3, 3
BCL10	1 (1)	1, 0
BCOR	1 (1)	0, 1
BCROL1	2 (3)	2, 0
CALR	1 (1)	0, 1
CBL	1 (1)	1, 0
CEBPA	1 (1)	1, 0
CSF1R	1 (1)	0, 1
CSF3R	1 (1)	1, 0
CSNK1A1	3 (4)	2, 1
DDX41	2 (3)	1, 1
DDX54	1 (1)	0, 1
DNMT3A	12 (17)	7, 5
EGFR	1 (1)	1, 0
EP300	2 (3)	0, 2
EZH2	6 (9)	3, 3
GATA2	3 (4)	1, 2
GNB1	1 (1)	0, 1
IDH1	1 (1)	1, 0
IDH2	1 (1)	1, 0
KMT2C	1 (1)	1, 0
KRAS	1 (1)	1, 0
LUC7L2	1 (1)	1, 0
NF1	3 (4)	1, 2
NRAS	2 (3)	1, 1
PPM1D	2 (3)	1, 1
PTPN11	2 (3)	1, 1
RAD50	1 (1)	1, 0
ROBO1	2 (3)	1, 1
RUNX1	1 (1)	1, 0
SF3B1	10 (14)	3, 7
SH2B3	2 (3)	1, 1
SRSF2	1 (1)	1, 0
TET2	3 (4)	0, 3
TP53	53 (76)	27, 26
TP53 1 mutation	4 (8)	2, 2
TP53 multi-mutations	49 (92)	25, 24
TP53 1 mutation + del	15 (31)	10, 5
TP53 1 mutation + cnloh	16 (33)	8, 8
TP53 >1 mutations	18 (37)	7, 11
TP53 WT	13 (19)	8, 5
ZBTB33	1 (1)	1, 0
No mutations	2 (3)	3, 1

Supplementary Table 7. Molecular response in responding patients

Patient N.	Diagnosis	Treatment	Response	Cytgenetic response	Total number of mutations	TP53 mutation	TP53 VAF%, inclusion			TP53 VAF%, final assessment			Other mutations at inclusion
							VAF 1	VAF 2	VAF 3	VAF 1	VAF 2	VAF 3	
9	MDS	AZA	CR	CR	2	multi-hit	0,398			0			RAD50
16	MDS	AZA+LEN	CR	CR	3	WT							BCOR, BCOR, DNMT3A
42	MDS	AZA+LEN	CR	CR	1	Single-mutation	0,536			0			
68	MDS	AZA	CR	CR	3	WT							BCORL1, PPM1D, IDH2
70	MDS	AZA	CR	CR	3	multi-hit	0,844			0			CSF3R, EZH2
17	MDS	AZA	CR	PR	2	multi-hit	0,71			0,06			DNMT3A
6	AML	AZA	marrow CR	CR	1	multi-hit	0,31						
23	MDS	AZA+LEN	marrow CR	CR	2	WT							EP300, SF3B1
29	MDS	AZA+LEN	marrow CR	CR	2	multi-hit	0,62			0			DNMT3A
33	MDS	AZA+LEN	marrow CR	CR	1	multi-hit	0,55			0			
67	AML	AZA+LEN	marrow CR	CR	4	WT							ASXL1, GATA2, PTPM11, PPM1D
72	MDS	AZA+LEN	marrow CR	CR	3	WT							SF3B1, GATA2, DDX41
25	MDS	AZA	marrow CR	PR	2	multi-hit	0,8			0,148			ASXL1
1	MDS	AZA+LEN	marrow CR	NR	2	multi-hit	0,396	0,398					
8	AML	AZA+LEN	marrow CR	NR	2	multi-hit	0,313						CSF1R
35	MDS	AZA	marrow CR	NR	3	multi-hit	0,112	0,044	0,117	0,054	0,077	0	
38	MDS	AZA	marrow CR	NR	1	multi-hit	0,622						
39	AML	AZA+LEN	marrow CR	NR	3	multi-hit	0,341	0,318		0	0	0,007	SF3B1
44	MDS	AZA	marrow CR	NR	1	multi-hit	0,864			0,27			
52	MDS	AZA	marrow CR	NR	4	multi-hit	0,264	0,289		0,22	0,228		EZH2, DNMT3A
60	MDS	AZA+LEN	marrow CR	NR	1	multi-hit	0,69						
61	MDS	AZA+LEN	marrow CR	NR	2	WT							SF3B1, CSNK1A1
5	MDS	AZA+LEN	HI	NR	2	multi-hit	0,71						EZH2
18	MDS	AZA+LEN	HI	NR	1	multi-hit	0,606			0,101			
19	MDS	AZA	HI	NR	2	multi-hit	0,261	0,26		0	0,052		
20	AML	AZA+LEN	HI	NR	3	multi-hit	0,369	0,35		0,076	0,065		DDX54
26	MDS	AZA	HI	NR	4	multi-hit	0,604			0,926			GATA2, NF1, PTPN11
51	MDS	AZA	HI		1	multi-hit	0,92						
55	MDS	AZA+LEN	HI	NR	0	del 17p but no mutation							
64	MDS	AZA	HI	NR	0	del 17p but no mutation							

Abbreviations: AML, acute myeloid leukemia; AZA, azacitidine; CR, complete remission; del, deletion; HI, hematologic improvement; LEN, lenalidomide; MDS, myelodysplastic syndrome; Mut, mutation; NR, no response; ORR, overall response rate; PR, partial remission; VAF, variant allele frequency, WT, wild type.

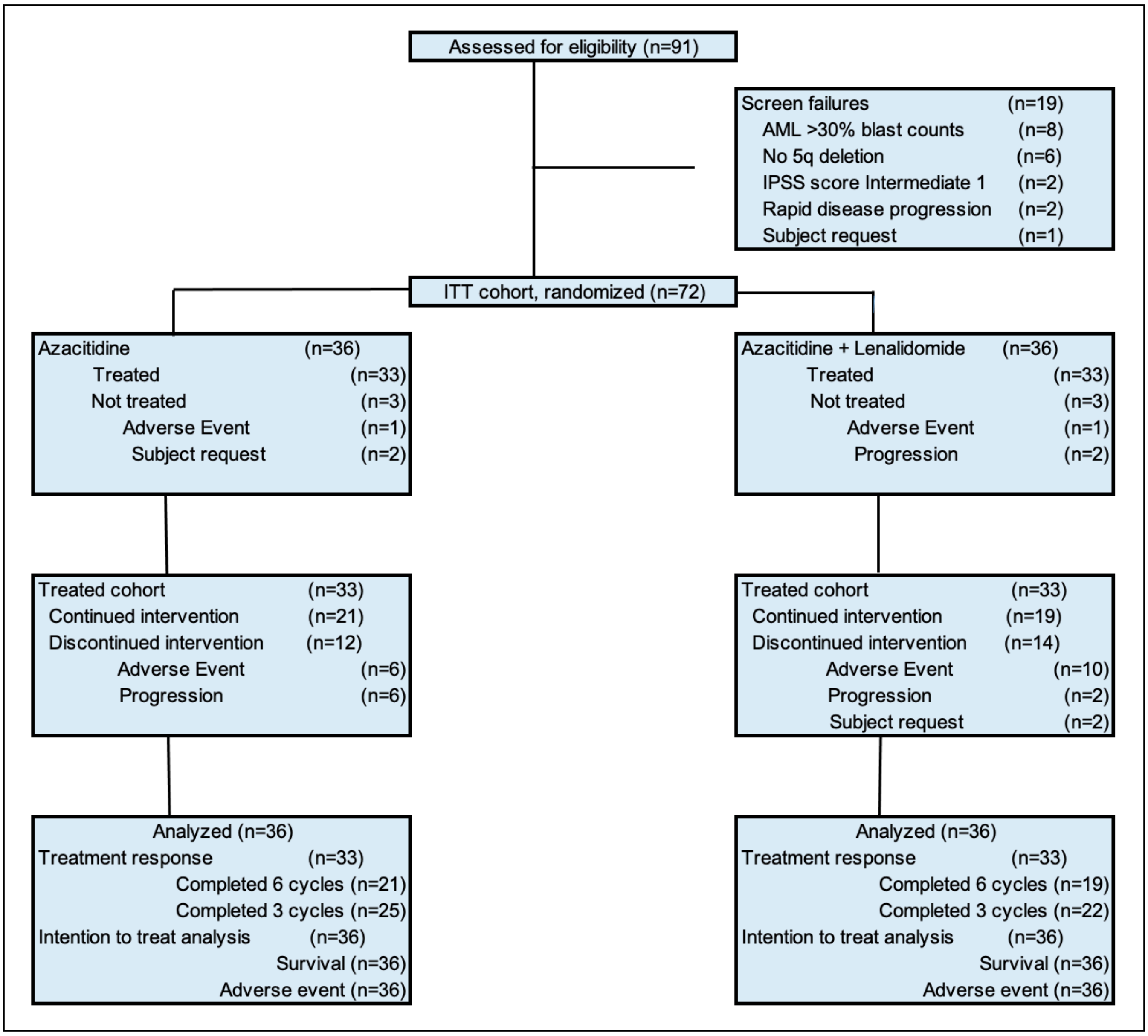

Supplementary Figure 1. CONSORT diagram. AML, acute myeloid leukemia; IPSS, International Prognostic Scoring System; ITT, intention to treat.

2a.

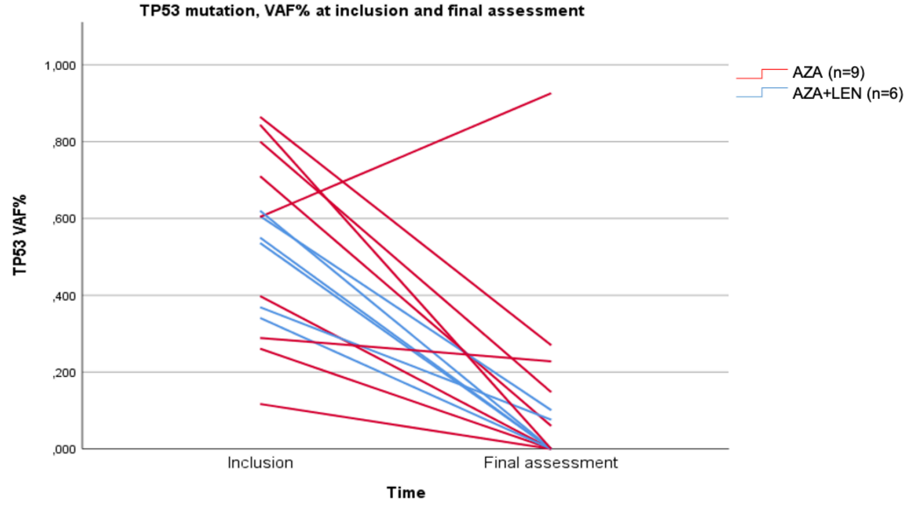

2b.

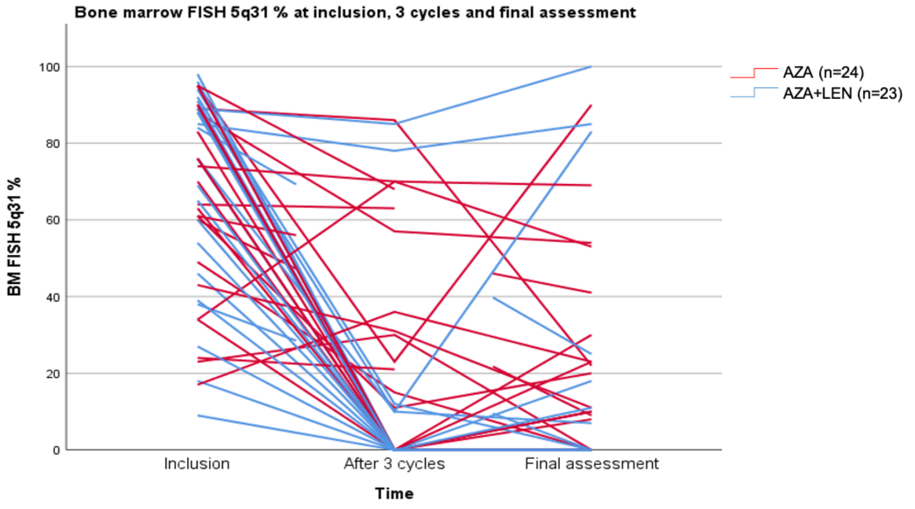

Supplementary Figure 2. Anti-tumor effect was analyzed by independent samples t-test. **2a.** TP53 variant allele frequency (VAF) % in responding patients at inclusion and at final assessment. No significant difference (P=0.49) between azacitidine (AZA) vs azacitidine + lenalidomide (AZA+LEN)**. 2b.** Bone marrow FISH 5q31 % at inclusion, after 3 cycles and final assessment. No significant difference between the two treatment arms, after 3 cycles (P=0.075) or at final assessment (P=0.67).
